# Transcriptome Analysis to Identify Crucial Genes for Reinforcing Flavins-Mediated Extracellular Electron Transfer in *Shewanella oneidensis*

**DOI:** 10.3389/fmicb.2022.852527

**Published:** 2022-06-01

**Authors:** Lixia Fang, Yuanyuan Li, Yan Li, Yingxiu Cao, Hao Song

**Affiliations:** ^1^Frontier Science Center for Synthetic Biology and Key Laboratory of Systems Bioengineering (Ministry of Education), School of Chemical Engineering and Technology, Tianjin University, Tianjin, China; ^2^Key Laboratory of Systems Bioengineering (Ministry of Education), Tianjin University, Tianjin, China

**Keywords:** *Shewanella oneidensis*, riboflavin, extracellular electron transfer, transcriptome analysis, ROS, heme accumulation

## Abstract

Flavins serve as the electron mediators in *Shewanella oneidensis*, determining the extracellular electron transfer (EET) rate. Currently, metabolic engineering of flavins biosynthetic pathway has been studied for improving EET. However, the cellular response triggered by flavins that contribute to EET remains to be elucidated. In this study, the riboflavin-overproducing strain C5 (expressing the flavins synthetic genes in plasmid PYYDT) and the PYYDT strain (harboring the empty plasmid PYYDT) in the microbial fuel cells are applied for comparative transcriptomic analyses to investigate beneficial gene targets that could improve EET. From the differentially expressed genes, we select the significantly upregulated and downregulated genes for inverse engineering in *S. oneidensis*. The results show that overexpression of *ahpC* and *ccpA*, and inactivation of *pubA*, *putB*, and *tonB* are able to improve the EET capability. Combinatorial modulation of these five genes results in the recombinant strain CM4, achieving the maximum power density of 651.78 ± 124.60 mW/m^2^, 1.97 folds of the parental strain. These genes modulation is speculated to reduce the ROS damage and to promote cytochrome synthesis and heme accumulation, which coherently enhance EET. Our findings facilitate in-depth understanding of the mechanism of flavins-mediated EET and provide new insights in promoting EET of *S. oneidensis* for electricity generation.

## Introduction

Extracellular electron transfer (EET) is the process that electroactive microorganisms (EAMs) acquire energy from the environment by extending respiratory chains to external electron acceptors (Beblawy et al., 2018; Chong et al., 2018; Light et al., 2018). EAMs, such as *Shewanella oneidensis*, are widely used as model microorganisms to study emerging bioelectrochemical technologies, including microbial fuel cell (MFC) (Lovley, 2006), microbial electrosynthesis (MES) (Rabaey and Rozendal, 2010), as well as pollutant degradation in bioremediation (Lu et al., 2016). It has been reported that approximately 70% of electrons from *S. oneidensis* cells to acceptors were transmitted by flavins, including flavin mononucleotide (FMN) and riboflavin (RF) (Marsili et al., 2008; Wu et al., 2013). The flavin molecules can act as diffusing electron shuttles and cytochromes-bound cofactors to mediate EET (Okamoto et al., 2014; Huang et al., 2018; Thirumurthy and Jones, 2020). The enhancement of electrochemically polymerized riboflavin interface could accelerate the interfacial electron exchange (Zou et al., 2018). Thus, the flavin molecules have a widespread contribution in microbial EET.

By engineering flavins biosynthetic pathways, EET could be greatly enhanced in *S. oneidensis* MR-1. For example, overexpression of the native flavin biosynthesis gene cluster increased the maximum current density in MFC by approximately 110% (Min et al., 2017). Besides, heterologous expression of the flavin biosynthesis pathway from *Bacillus subtilis* resulted in a 13.2-fold increase in the maximum power output (Yang et al., 2015). On this basis, flavins biosynthesis was further fine-tuned by different promoters and ribosome binding sites, which obtained an increase of 20% in the maximum power density (Lin et al., 2018). These studies successfully improved EET capacity through strengthening the flavin biosynthesis alone. However, numerous cellular responses for flavin-dependent EET remain unclear, which limits further improvement of electron transfer in *S. oneidensis*.

Flavin-mediated EET is a sophisticated process that involves many cellular processes, including intracellular substrates, available energy, and regulatory interactions. On the one hand, flavin molecules act as diffusing electron shuttles and transfer electrons through the transformation of oxidation and reduction state (Ross et al., 2011), which is correlated with a series of intracellular redox reactions. For example, increasing releasable electrons and available NADH promote electron transmission (Li et al., 2018). On the other hand, flavins also serve as cytochromes-bound cofactors to mediate extracellular electron transport (Okamoto et al., 2014; Huang et al., 2018; Thirumurthy and Jones, 2020). The interactions between cytochromes c and flavins as well as the EET-related pathways are tightly regulated by some global factors under the control of complicated cellular metabolism. For example, elevating the levels of intracellular second messenger cyclic AMP (cAMP) enabled to coordinately upregulate the expression levels of the genes encoding the C-type cytochromes (*c*-Cyts) and flavins synthetic pathways (Cheng et al., 2020). In addition, flavin-mediated EET interacts closely with other cellular processes and physiologies. Flavins synthesis may place cells under nutrition or redox stresses, and cellular responses must occur to maintain cellular viability and functions (Bankapalli et al., 2015). As such, it is of great importance to investigate beneficial cellular perturbation and metabolic mechanisms for improving flavins-mediated EET.

Here, we explore beneficial gene targets by transcriptome analysis and strengthen the favorable cellular responses to improve flavin-mediated EET. We identified five novel targets and confirmed their functions for improving EET by inverse engineering and electrochemistry analysis. As a result, the overexpression of *ahpC* and *ccpA* as well as the inactivation of *pubA*, *putB*, and *tonB* improved flavins-mediated EET. The underlying mechanism may be relevant to the decrease in reactive oxygen species (ROS) and the accumulation of heme for strengthening electron transfer. The engineered strain with combinatorial modulation of the identified five genes achieved the maximum power density of 651.78 ± 124.60 mW/m^2^, which was 1.97 folds of the parental strain. Our findings provide an increased understanding of cellular responses and metabolic rewiring that facilitate EET.

## Materials and Methods

### Enzymes and Chemicals

Enzymes (Phanta Max Super-Fidelity DNA polymerase, FastDigest restriction enzymes, and T4 DNA ligase) were purchased from Thermo Fisher Scientific (Shanghai, China). Kits for DNA purification, plasmid and genome extraction were supplied by TianGen (Beijing, China). All chemical reagents were purchased from Dingguo Changsheng Biotech (Tianjin, China). FAD, FMN, and RF were purchased from Sigma–Aldrich (Shanghai, China).

### Strains, Plasmids, and Growth Conditions

RF-overproducing *S. oneidensis* strain, namely C5 (Yang et al., 2015), was constructed previously and used as the base strain in this study. The plasmid PHG13 was used for gene expression (Cao et al., 2019). The strains and plasmids used in this study are listed in Table 1. For plasmid propagation, *E. coli* Trans1 T1 was grown in Luria-Bertani (LB) medium. For conjugation, strain *E. coli* WM3064 was cultivated in LB supplemented with 100 μg/mL 2, 6-diaminopimelic acid (DAP). *E. coli* strains were grown at 37°C with shaking at 220 rpm. *S. oneidensis* and derivative strains were cultivated in LB or anode solution (including M9 buffer, LB medium, MgSO_4_, CaCl_2_, and 20 mM lactate) at 30^°^C with shaking at 200 rpm. When required, antibiotics were added to a final concentration with 50 μg/mL kanamycin and 34 μg/mL chloramphenicol.

**TABLE 1:** Strains and plasmids used in this study.

Strains and plasmids	Characteristics	Sources
*S. oneidensis* MR-1	Wild type	Our lab
*E. coli* WM3064	A DAP auxotroph of *E. coli* could transfer plasmid into *S. oneidensis* MR-1 by conjugation	Our lab
*E. coli* trans1 T1	Cloning strain	Our lab
PYYDT	pTac, *rep^pBBR^*^1^, KanR, *oriT*	Yang et al., 2015
PYYDT-C5 (C5)	Wild type harboring PYYDT-*ribA-ribD-ribE-ribH-ribC*	Yang et al., 2015
PHG13-CB	pBad, *rep^ColE^*, CmR, *oriT*	Cao et al., 2019
PHG13-CT	pTet, *rep^ColE^*, CmR, *oriT*	Cao et al., 2019
PHG13-CL	placUV5, *rep^ColE^*, CmR, *oriT*	Cao et al., 2019
PHG13-CB-gene	pBad, *rep^ColE^*, CmR, *oriT*, *gene*	This study
PHG13-CT-gene	pTet, *rep^ColE^*, CmR, *oriT*, *gene*	This study
PHG13-CL-gene	placUV5, *rep^ColE^*, CmR, *oriT*, *gene*	This study
DT0	*S. oneidensis* MR-1 harboring C5 and PHG13-CT	This study
DT1	*S. oneidensis* MR-1 harboring C5 and PHG13-CT-*ccpA*	This study
DT2	*S. oneidensis* MR-1 harboring C5 and PHG13-CT-*ahpF*	This study
DT3	*S. oneidensis* MR-1 harboring C5 and PHG13-CT-*ahpC*	This study
DB0	*S. oneidensis* MR-1 harboring C5 and PHG13-CB	This study
DB1	*S. oneidensis* MR-1 harboring C5 and PHG13-CB-*ccpA*	This study
DB2	*S. oneidensis* MR-1 harboring C5 and PHG13-CB-*ahpF*	This study
DB3	*S. oneidensis* MR-1 harboring C5 and PHG13-CB-*ahpC*	This study
DL0	*S. oneidensis* MR-1 harboring C5 and PHG13-CL	This study
DL1	*S. oneidensis* MR-1 harboring C5 and PHG13-CL-*ccpA*	This study
DL2	*S. oneidensis* MR-1 harboring C5 and PHG13-CL-*ahpF*	This study
DL3	*S. oneidensis* MR-1 harboring C5 and PHG13-CL-*ahpC*	This study
DL4	*S. oneidensis* MR-1 harboring C5 and PHG13-CL-*ahpC*-*ccpA*	This study
BE1	Inactivated gene *pubA*	This study
BE2	Inactivated gene *pubB*	This study
BE3	Inactivated gene *pubC*	This study
BE4	Inactivated gene *putA*	This study
BE5	Inactivated gene *putB*	This study
BE6	Inactivated gene *hmuA*	This study
BE7	Inactivated gene *tonB*	This study
BE8	Inactivated gene *exbB*	This study
BE9	Combination of inactivated gene *putB, pubA*, and *tonB*	This study
CM1	Combinational modulation of DL3 and BE7	This study
CM2	Combinational modulation of DL3 and BE9	This study
CM3	Combinational modulation of DL4 and BE7	This study
CM4	Combinational modulation of DL4 and BE9	This study

### Construction of Plasmids

The sequences of primers used for PCR amplification are listed in Supplementary Table 1. The genes *ccpA*, *ahpF*, and *ahpC* were amplified using corresponding primer pairs *ccpA*-F/R, *ahpF*-F/R, and *ahpC*-F/R with *S. oneidensis* MR-1 genome as a template. The resulting fragments and plasmids PHG13-CB, PHG13-CT, and PHG13-CL were digested with the restriction endonuclease *Nde*I/*Nhe*I, followed by ligation, leading to PHG13-CB-gene, PHG13-CT-gene, and PHG13-CL-gene, respectively. Genetic assembly of *ahpC* and *ccpA* was constructed by BioBrick standards (Cao et al., 2019). Plasmid PHG13-CL-*ccpA* was digested with *Avr*II and *Nhe*I, then ligated into the plasmid PHG13-CL-*ahpC* that was digested by *Nhe*I, resulting in plasmid PHG13-CL-*ahpC*-*ccpA*.

### CRISPR Base Editing

The CRISPR base editing mediated by dCas9 and activation-induced cytidine deaminase (AID) has a 5 nt target window that all C within −16 to −20 positions upstream of the PAM sequence can be edited to T, which results in the stop codons (TAG, TAA, and TGA) (Komor et al., 2016; Kim et al., 2017). For CRISPR/dCas9-AID genome editing in *S. oneidensis*, dCas9-AID and sgRNA in plasmid 104 were expressed to obtain the desirable mutations (Supplementary Figure 1). To construct sgRNA expression plasmids, the pairs of 24-bp primers (Supplementary Table 2) were annealed to form a double-strand DNA with cohesive ends, which were ligated to *Bsa*I sites in plasmid 104 using Golden Gate assembly (Wang et al., 2018).

*S. oneidensis* strains transformed with the sgRNA expression plasmids were plated on LB-agar plates supplemented with 0.8 mM IPTG and 50 μg/mL kanamycin. Plates were incubated 18–24 h at 30^°^C until colonies were visible. For the complete base mutation, colonies in plates were incubated in LB-liquid medium with IPTG and kanamycin for 12 h. For plasmid curing, cells were transferred to LB-liquid culture without antibiotic and incubated overnight at 30^°^C. Subsequently, cell suspensions were streaked on fresh LB-agar plates and incubated overnight to generate single colonies. These colonies were plated on LB plates containing kanamycin and incubated at 30°C to test them for plasmid loss.

### Tube Fermentation for Riboflavin Production

Fermentation was performed as follows: a cryopreservation stock stored at −80°C was streaked on fresh LB-agar plates and incubated at 30°C until colonies were visible. After that, the colonies on the plate were suspended in 3 mL LB medium and incubated for 12 h to acquire the seed suspension, which was used to inoculate 30 mL test tubes containing 5 mL of the fermentation medium, adjusting the initial OD_600_ to 0.01. The fermentation medium was LB medium supplemented with 20 mM lactate and corresponding antibiotics depending on plasmids. The inducers were added at the early exponential phase (OD_600_ = 0.5–0.6) with corresponding concentrations (0.75 mM IPTG for promoter placUV5, 10 mM arabinose for promoter pBad, and 1,000 ng/ml anhydrotetracycline hydrochloride for promoter pTet). The fermentation was conducted by inducing for 12 h, and the cell growth and the RF production were measured subsequently.

### High Performance Liquid Chromatography Analysis of Flavins

The production of extracellular RF was measured by using High Performance Liquid Chromatography (HPLC) with a UV detector (Shimadzu, Japan). Fresh samples of cell cultures were harvested by centrifugation, and all standard solutions and sample supernatants were filtered using a 0.45 μm syringe filter, and then assayed by a reverse phase C18 column (5 μm particle size, 250 mm × 4.6 mm, Thermo Fisher Scientific). The column was equilibrated into 30% (v/v) methanol and NaH_2_PO_4_, an isocratic eluent was applied over 25 min at a flow rate of 0.6 mL/min at 30°C with 20 μL injection volume. The range of concentration for standard solutions were 1, 10, 50, 100, 200, and 400 mg/L. The elution times were around 7.2 min for FAD, 11.9 min for FMN, and 19.1 min for RF, respectively.

### Microbial Fuel Cell Setup

To evaluate the efficiency of EET, the overnight *S. oneidensis* culture suspension (0.5 mL) was inoculated into 50 mL fresh LB medium supplemented with corresponding antibiotics and IPTG. After incubated for 12 h, the cell suspension concentration was adjusted OD_600_ to 0.5 and dispersed into the bioanode of MFCs. When required, 50 μg/mL kanamycin and 1 mM IPTG were added in anolyte to ensure consistent culture conditions. All MFCs were cultivated in a 30°C incubator and each group was tripled for parallel experiments.

Dual-chamber MFCs with a working volume of 110 mL were applied in this study. Carbon cloth (CeTech, China) was used as the electrodes for both anode (1.0 cm × 1.0 cm) and cathode (2.5 cm × 3 cm). The Nafion 117 membrane (DuPont Inc., United States) was used to separate the anode and cathode. The anolyte consisted of M9 buffer (Na_2_HPO_4_, 42.3 mM; KH_2_PO_4_, 22.1 mM; NaCl, 8.5 mM; NH_4_Cl, 18.7 mM; MgSO_4_, 1 mM; CaCl_2_, 0.1 mM), supplemented with 20 mM lactate and 5% (v/v) LB broth. The cathodic electrolyte was composed of 50 mM K_3_[Fe (CN)_6_], 50 mM KH_2_PO_4_, and 50 mM K_2_HPO_4_. Besides, to measure the voltage generated, a 2 kΩ external resistor was connected to the external circuit of MFCs.

### Bioelectrochemical Analyses

The voltage output was recorded using a digital multimetera (DT9205A). The cyclic voltammetry (CV) curve and linear sweep voltammetry (LSV) curve were conducted during the plateau of voltage output of MFC. CV curve was performed in a three-electrode configuration included an Ag/AgCl reference electrode (Aida Hengsheng Co., China) with a 1 mV/s scan rate in the range from −0.7 to 0.1 V. Polarization curve and power density curve were obtained by LSV which was decreasing the potential at a rate of 0.1 mV/s from OCP (open circuit potential). Both the CV curve and LSV curve were monitored by an electrochemical workstation CHI1000C (CH Instrument, Shanghai, China).

### Transcriptome Analysis

The planktonic cells were harvested after 4 or 12 h of cultivation in MCFs by quick centrifugation at 10,000 × g for 1 min and immediately frozen in liquid nitrogen. Total RNA was extracted using the RNAprep pure Cell/Bacteria Kit (Tiangen) following lysozyme treatment. RNA degradation and contamination were monitored on 1% agarose gels. RNA integrity was assessed on a 2100 Bioanalyzer (Agilent Technologies, CA, United States). RNA concentration was measured by Qubit^®^ RNA Assay Kit in Qubit^®^ 2.0 Flurometer (Life Technologies, CA, United States). rRNA was removed using a Ribo-zero kit that left the mRNA. A total of 3 μg RNA was used as an input per sample. Sequencing libraries were generated using a NEBNext^®^ Ultra™ RNA Library Prep Kit for Illumina^®^ (NEB, United States) following the manufacturer’s instructions. Index codes were added to attribute sequences to each sample. According to the manufacturer’s instructions, clustering of the index-coded samples was performed on a cBot Cluster Generation System using the TruSeq PE Cluster Kit v3-cBot-HS (Illumina). The library preparations were sequenced on an Illumina Hiseq 4000 platform and paired-end reads were generated. The data were analyzed by Beijing Novogene Bioinformatics Technology Co., Ltd. (China). Specifically, raw reads were firstly processed through in-house perl scripts, generating clean data with high quality. The index of reference genome was built and clean reads were aligned to reference genome^1^ by using Bowtie2 v2.2.3. HTSeq v0.6.1 was used to count the read numbers mapped to each gene. Then, differential expression analysis of the C5 strain compared with the PYYDT strain was performed using the DEGSeq v1.12.0. *p*-values were adjusted using the Benjamini and Hochberg method and corrected to *q*-value. Lists of differentially expressed genes [abs (log_2_ fold change) > 1 and *q*-value < 0.005] in the C5 strain compared with the PYYDT strain at 4 and 12 h are finally presented in Data Sheet 1 and Data Sheet 2, respectively.

## Results and Discussion

### Transcriptional Profiling of Riboflavin-Overproducing *Shewanella oneidensis*

**FIGURE 1:**
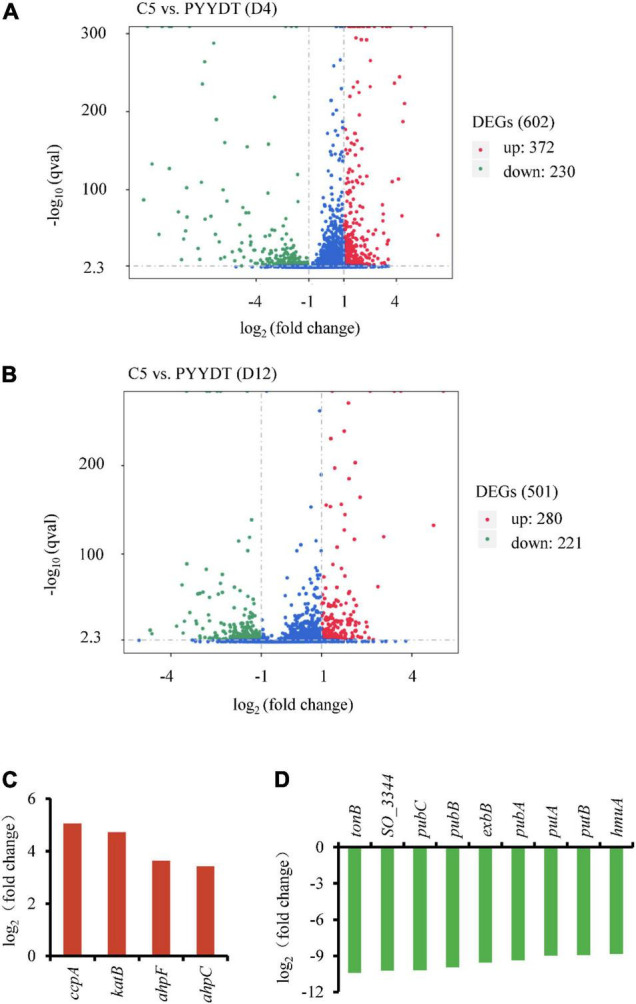
Comparative transcriptome analysis of the C5 and reference strains. **(A,B)** Differentially expressed genes in the C5 strain compared with the reference strain at 4 h **(A)** and 12 h **(B)**. Red dots represent the upregulated genes [log_2_ (fold change) > 1 and *q*-value < 0.005], and the green dots represent the downregulated genes [log_2_ (fold change) < −1 and *q-*value < 0.005]. **(C,D)** Expression levels of significantly upregulated genes at 12 h **(C)** and downregulated genes at 4 h **(D)**. All of these genes were applied for the next inverse engineering.

The engineered *S. oneidensis* strain C5 expressing the RF biosynthetic genes (*ribA*, *ribD*, *ribE*, *ribH*, and *ribC*) in plasmid PYYDT exhibited a dramatically improved EET capability in MFC, and the maximum power density reached 233.0 ± 24.9 mW/m^2^, which was 14.2 folds of the PYYDT strain that transformed with an empty vector PYYDT (Yang et al., 2015). To exploit beneficial cellular responses associated with flavins-mediated EET, we performed a comparative transcriptome analysis of the C5 and the PYYDT strains. Cells incubated in MFC were sampled at 4 and 12 h, the early and middle exponential phase of the output voltage (Supplementary Figure 2). As shown in Figure 1A, there were 602 differently expressed genes in the C5 strain compared with the PYYDT strain at 4 h, including 372 upregulated genes [log_2_ (fold change) > 1 and *q*-value < 0.005)] and 230 downregulated genes [log_2_ (fold change) < −1 and *q*-value < 0.005] (Data Sheet 1). There were 501 differently expressed genes in the C5 strain compared with the PYYDT strain at 12 h, including 280 upregulated genes [log_2_ (fold change) > 1 and *q*-value < 0.005] and 221 downregulated genes [log_2_ (fold change) < −1 and *q*-value < 0.005] (Figure 1B, Data Sheet 2). The results indicated that in the C5 strain, higher EET capability may be affected not only by the engineering of flavin synthetic pathway but also secondary cellular responses to RF overproduction.

To investigate potential gene targets associated with the RF-dependent EET, we evaluated the effect of their overexpression/inactivation on the production of electricity. Considering the process of gene reverse-engineering, we selected downregulated and upregulated targets at an earlier (4 h) and later (12 h) time, respectively. The top four upregulated genes at 12 h included *ccpA, katB*, *ahpF*, and *ahpC* (Figure 1C). Gene *ccpA* encodes a peroxidase that involves in the metabolism of *c*-Cyts. Gene *katB* encodes the catalase that responses to hydrogen peroxide. Genes *ahpC* and *ahpF* are relevant to cellular redox reactions (Seaver and Imlay, 2001). Additionally, the top nine downregulated genes at 4 h included *tonB*, SO_3344, *pubC*, *pubB*, *exbB*, *pubA*, *putA*, *putB*, and *hmuA* (Figure 1D). All of these downregulated genes are involved in the process of extracellular iron uptake (Huang and Wilks, 2017). However, the EET capability of these differentially expressed genes has not been investigated in previous studies. Consequently, it is necessary to modulate these gene targets to validate whether the reinforcement of cellular response could enhance the flavins-mediated EET.

### Exploration of Beneficial Upregulated Targets for Improving Extracellular Electron Transfer Capability

**FIGURE 2:**
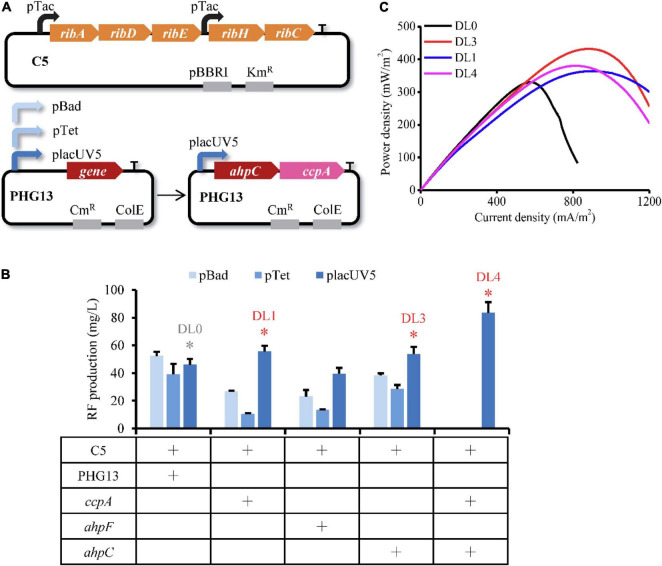
Inverse engineering of the selected upregulated genes for evaluating EET performance. **(A)** Schematic illustration of dual plasmids expression system used for inverse engineering of the selected genes. Target genes were overexpressed with the pBad, pTet, or placUV5 promoters, respectively. Genes *ahpC* and *ccpA* were co-expressed by the placUV5 promoter. **(B)** RF production of the engineered strains overexpressing the selected genes. The red asterisks represent the strains with increased RF production compared with the corresponding reference strain (in gray asterisk), which were used for electrochemical analysis. The error bars (mean ± SD) were derived from triplicate experiments for each strain. **(C)** Power density output curves of the selected strains in MFC.

To evaluate if overexpression of the upregulated targets can increase EET, we expressed each of these genes by a second plasmid PHG13 in the C5 strain (Figure 2A). To avoid the metabolic burden caused by two plasmids and inappropriate expression intensity, expression of the upregulated genes was fine-tuned by different promoters, including the high-strength pBad promoter, the medium-strength pTet promoter, and the low-strength placUV5 promoter (Cao et al., 2019). Gene *katB* was failed to be amplified from the genome. Considering the importance of flavin in EET, we firstly assayed RF titer of the engineered strains. Tube fermentation was conducted in LB medium supplemented with 20 mM lactate for 12 h after induction. Cell growth of the engineered strains was measured (Supplementary Figure 3). As shown in Figure 2B, overexpression of the upregulated genes driven by the high-strength pBad or medium-strength pTet promoters severely reduced RF production, while their expression controlled by the low-strength placUV5 promoter had no adverse effect on the RF production. The DL0 strain produced slightly decreased RF compared to the C5 strain (Supplementary Figure 4), which might due to the expression of the empty plasmid PHG13. Particularly, the DL1 strain (overexpressing *ccpA* by placUV5 promoter in C5 strain) and the DL3 strain (overexpressing *ahpC* by placUV5 promoter in C5 strain) exhibited higher RF production than the DL0 strain (overexpressing empty plasmid PHG13 in C5 strain). Furthermore, these two targets were co-expressed and the resulted DL4 strain obtained a further enhanced RF production (Figures 2A,B).

To investigate the effect of these upregulated targets on electron transfer, the recombinant strains DL1, DL3, and DL4 were inoculated in anodic chambers of MFCs for measuring the voltage output. The linear sweep voltammetry (LSV) was then performed during the plateau of voltage to measure the power density. As shown in Figure 2C, the maximum power densities of the DL3, DL1, and DL4 strains reached 432.18 ± 131.18, 364.09 ± 16.20, and 380.22 ± 55.05 mW/m^2^, respectively, which were 1.31, 1.10, and 1.15 folds of the DL0 strain. These results indicated that overexpression of genes *ahpC* and *ccpA* controlled by the low-strength placUV5 promoter further facilitated RF-mediated EET.

### Investigation of Beneficial Downregulated Targets for Enhancing Extracellular Electron Transfer Efficiency

**FIGURE 3:**
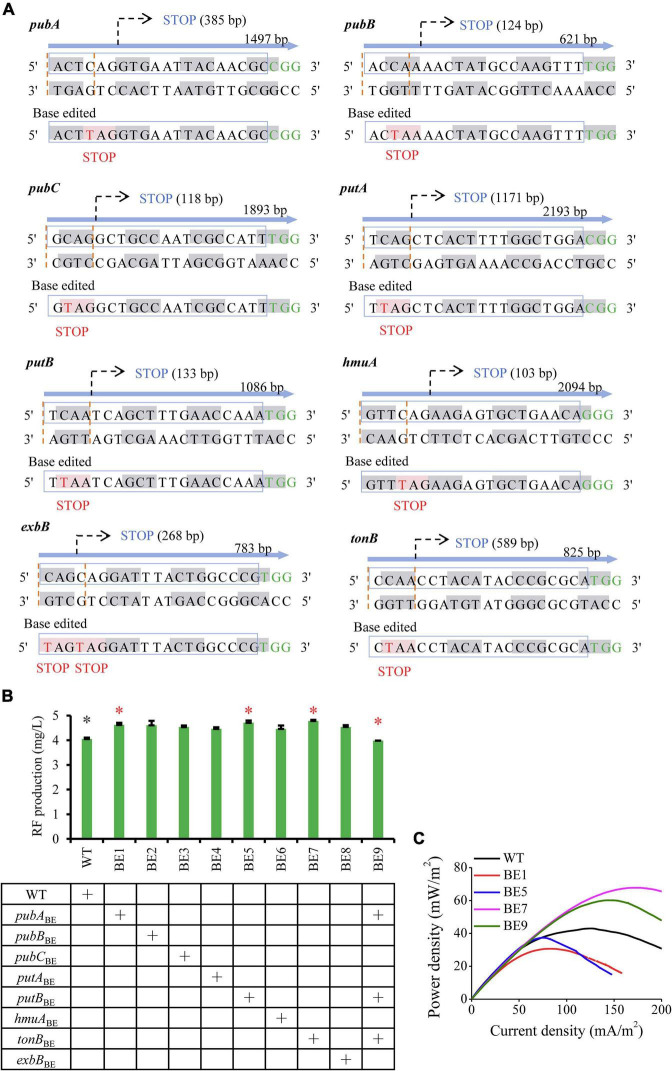
Inverse engineering of the selected downregulated genes for evaluating EET performance. **(A)** Base editing events of significantly downregulated genes. Codon nucleotides are indicated by gray and white alternately. The target site of sgRNA is indicated by the blue box and the PAM sequences are highlighted in green. The expected editing codon nucleotides are indicated with “STOP” upstream of the PAM sequence and the edited base is highlighted in red. **(B)** RF production of engineered strains that inactivated the selected genes. Strains labeled with asterisks were used for assessing EET, including the engineered strains (red asterisks) and the reference strains (gray asterisks). The error bars (mean ± SD) were derived from triplicate experiments for each strain. **(C)** Power density output curves of the selected strains in MFCs.

To assess the effect of downregulated targets on EET, genes *pubB, pubC, pubA, tonB, exbB, hmuA, putA*, and *putB* (Figure 1C) were inactivated through CRISPR base editing. CRISPR/Cas9-AID converts C base to T base and induces nonsense mutations (Wang et al., 2018). The inactivated sites of the selected genes were shown in Figure 3A. Gene SO_3344 was failed to design the inactivated sites. To evaluate whether these gene inactivations impair cell growth and RF metabolism, tube fermentation of the engineered strains was performed in LB medium with 20 mM lactate for 12 h. As shown in Figure 3B and Supplementary Figure 3, modification of these genes did not decrease RF production and cell growth. Particularly, the BE1 (inactivating *pubA*), BE5 (inactivating *putB*), and BE7 (inactivating *tonB*) strains obtained increased RF production. Genes *pubA*, *putB*, and *tonB* were further combinatorically inactivated, resulting in the BE9 strain. The combination of these genetic perturbations has no negative effect on cell growth (Supplementary Figure 5) and exhibits only a slightly decreased RF production (Figure 3B).

The BE1, BE5, BE7, and BE9 strains were applied for further detection of electrochemical characteristics. These engineered strains were inoculated in anodic chambers of MFCs and the voltage output was measured. The LSV was then performed during the plateau of voltage to measure the power density. As shown in Figure 3C, compared to the wild-type strain (WT), the BE1 and BE5 strains showed reduced electron transfer capability, while the BE7 and BE9 strains exhibited improved EET capability. The maximum power densities of the BE7 and BE9 strains reached 74.78 ± 4.93 and 62.19 ± 4.35 mW/m^2^, which were 1.72 and 1.43 folds of the WT strain, respectively. These results confirmed that simultaneous inactivation of gene targets *tonB*, *pubA*, and *putB*, or inactivation of *tonB*, can greatly accelerate the electron transfer in *S. oneidensis*.

### Speculation of the Mechanism of Riboflavin-Mediated Extracellular Electron Transfer

Our studies indicated that overexpression of *ahpC* and *ccpA* efficiently improved the EET capability of *S. oneidensis* (Figure 2C). To exploit the corresponding mechanism, we analyzed the functions of beneficial genes. A previous study indicated that the increased RF was prone to generate destructive ROS (Xu et al., 2015), which resulted in severe DNA damage and impaired cell viability (Imlay, 2003). Gene *ahpC*, encoding the alkyl hydroperoxide reductase peroxiredoxin, enables scavenging the intracellular ROS (Seaver and Imlay, 2001). Likewise, gene *ccpA*, encoding the periplasmic c-type cytochrome peroxidase, has been reported to catalyze the reduction of hydrogen peroxide, which results in detoxification of ROS (Hoffmann et al., 2009; Schutz et al., 2011). Consequently, we speculated that upregulation of *aphC* and *ccpA* could provide a favorable physiological status for EET.

Hemin in *c*-Cyts can be reduced through the oxidation of RF (Wang et al., 2017), which facilitates the generation of heme and then enhances EET capability. We found that inactivation of genes *tonB*, *pubA*, and *putB* also improved the electricity production of *S. oneidensis* (Figure 3C). TonB is an energy transducer, which is necessary to transport extracellular iron into intracellular (Minandri et al., 2016; Balhesteros et al., 2017). The siderophore putrebactin, encoded by gene *pubA*, involves ferric iron uptake (Kadi et al., 2008). The reductase encoded by *putB* can catalyze the generation of ferrous iron (Liu et al., 2018). Therefore, we speculate that the inactivation of *tonB*, *pubA*, and *putB* hinders ferric iron entering into cytoplasm and increases its extracellular availability. The increase of ferric iron could facilitate hemin accumulation and accelerate its conversion to heme, which promotes the transfer of the intracellular electron (Kranz et al., 2009).

### Combinatorial Perturbation of Gene Targets for Improving Extracellular Electron Transfer

**FIGURE 4:**
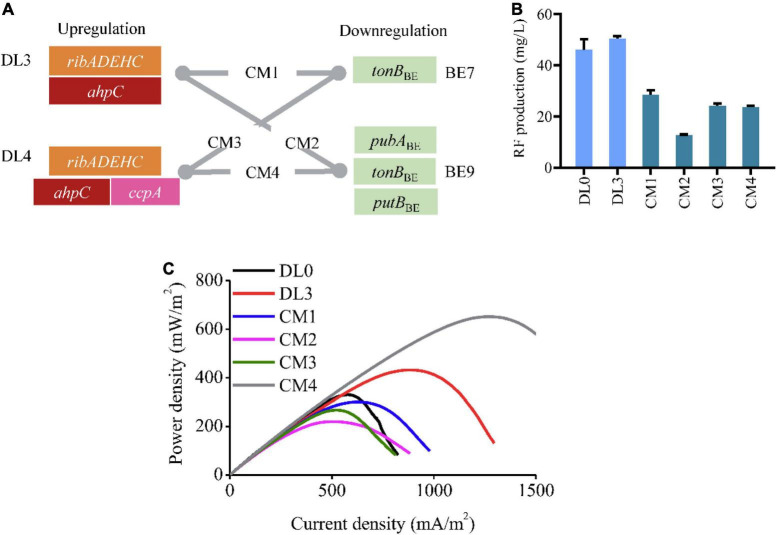
Combinational modulation of the identified genes for improving EET. **(A)** Schematic illustration of combinatorial perturbation in the engineered strains. The combination of gene perturbation in the DL3 (expressing C5 and *ahpC*) or DL4 (expressing C5, *ahpC*, and *ccpA*) strains with that in the BE7 (inactivating *tonB*) or BE9 (inactivating *tonB*, *pubA*, and *putB*) strains resulted in four recombinant strains CM1, CM2, CM3, and CM4, respectively. **(B)** RF production of the combinatorial modulation strains (CM1, CM2, CM3, and CM4). The error bars (mean ± SD) were derived from triplicate experiments for each strain. **(C)** The power density of the combinatorial modulation strains (CM1, CM2, CM3, and CM4).

In the above study of exploration of beneficial genes, the DL3 (expressing *ahpC* in C5 strain) and DL4 (expressing *ahpC* and *ccpA* in C5 strain) strains as well as the BE7 (inactivating *tonB*) and BE9 (inactivating *tonB*, *pubA*, and *putB*) strains obtained improved power density. To further accelerate the electron transfer, we combined gene perturbation in these strains and resulting in four recombinant strains, CM1 (expressing *ahpC* and inactivating *tonB* in C5 strain), CM2 (expressing *ahpC* and inactivating *tonB*, *pubA*, and *putB* in C5 strain), CM3 (expressing *ahpC*, *ccpA*, and inactivating *tonB* in C5 strain), and CM4 (expressing *ahpC*, *ccpA*, and inactivating *tonB*, *pubA*, and *putB* in C5 strain) (Figure 4A). We firstly assayed RF production of the engineered strains, and found that the combined gene perturbation reduced the RF titer compared to the DL0 strain (Figure 4B). We then conducted LSV to evaluate the electrochemical characteristics of these recombinant strains. The maximum power density of the CM4 strain reached 651.78 ± 124.60 mW/m^2^, which was 1.97 and 1.51 folds of the DL0 and DL3 strains, respectively (Figure 4C). The results demonstrated that the combination of upregulating *ahpC* and *ccpA* and inactivating *tonB*, *pubA*, and *putB* significantly enhanced flavin-mediated EET capability in *S. oneidensis*.

EET is a sophisticated network that involves the coordination of multiple intermediates and complex interactions (Lubner et al., 2017; Ikeda et al., 2021). Thus, many in-depth targets might also lead to unexpected improvement for the desired phenotype. Transcriptome analysis has provided an engineering blueprint for metabolic rewiring and enabled unveiling non-intuitive key targets in the process of cellular metabolism (Ajjawi et al., 2017; Fang et al., 2021), which facilitate for exploring underlying mechanisms on cellular responses (Huang et al., 2017; Liu et al., 2019). In this study, we utilized transcriptome analysis to investigate beneficial gene targets in RF-producing strain. We found that the MtrABC complex genes were upregulated at the transcript level in the C5 strain compared with the PYYDT strain, while the transcript of riboflavin biosynthetic genes (*ribA*, *ribD*, *ribE*, and *ribC*) changed slightly (Supplementary Table 3). The results suggested the enhanced flavin-mediated EET capability in the C5 strain. We identified five novel targets *ahpC*, *ccpA*, *putB*, *pubA*, and *tonB* that were beneficial to flavins-dependent EET. Notably, these genes have not been engineered for EET in previous studies. Overexpression of *ahpC* and *ccpA* were speculated to reduce the destructive ROS. Inactivation of *putB*, *pubA*, and *tonB* might increase heme availability, which accelerate the electron transmission. Thus, we identified novel clues for improving power density and elucidating the mechanism of RF-mediated EET.

## Conclusion

To exploit the flavins-mediated EET mechanism and enhance the EET capability in *S. oneidensis*, we utilized transcriptome analysis for identifying efficient gene targets in RF-overproducing strain C5. We found that the overexpression of *ahpC* and *ccpA*, as well as inactivation of *putB*, *pubA*, and *tonB*, could reinforce the flavins-mediated EET. Particularly, the recombinant CM4 strain (expressing *ahpC*, *ccpA*, and inactivating *tonB*, *pubA*, and *putB* in C5 strain) obtained the maximum power density of 651.78 ± 124.60 mW/m^2^, 1.97 folds of the DL0 strain. Our findings contribute to further understanding the mechanism of EET and provide new insights in promoting flavins-mediated EET in *S. oneidensis*.

## Data Availability Statement

The original contributions presented in the study are included in the article/Supplementary Material, further inquiries can be directed to the corresponding author/s.

## Author Contributions

LF, YYL, YC, and HS conceived, designed the experiments, and wrote the manuscript. YYL and YL performed the plasmids construction experiments, tube fermentation experiments, and bioelectrochemical analyses. LF, YYL, and YC analyzed the data. YC and HS supervised all aspects of the study. All authors contributed to the article and approved the submitted version.

## Conflict of Interest

The authors declare that the research was conducted in the absence of any commercial or financial relationships that could be construed as a potential conflict of interest.

## Publisher’s Note

All claims expressed in this article are solely those of the authors and do not necessarily represent those of their affiliated organizations, or those of the publisher, the editors and the reviewers. Any product that may be evaluated in this article, or claim that may be made by its manufacturer, is not guaranteed or endorsed by the publisher.

## Footnotes

1
https://www.ncbi.nlm.nih.gov/nuccore/AE014299.2?report=genbank
