# Supplementary material for: Transcriptome Analysis to Identify Crucial Genes for Reinforcing Flavins-Mediated Extracellular Electron Transfer in Shewanella oneidensis
Source: Front Microbiol. 2022 Jun 1;13:852527. doi: 10.3389/fmicb.2022.852527 (PMC9198578; doi:10.3389/fmicb.2022.852527)
Supplement: Supplementary file 3 [file Data_Sheet_3.docx]

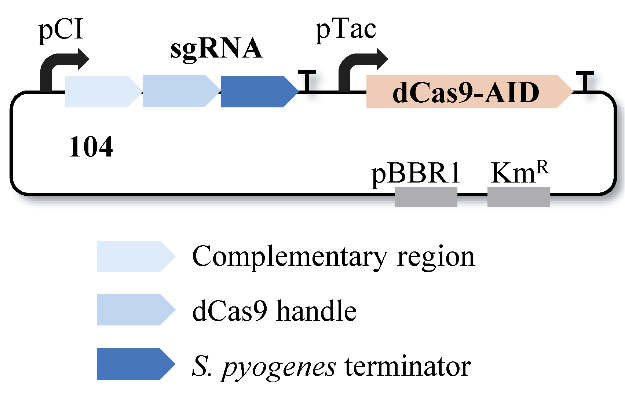


**Supplementary Figure 1.** Schematic illustration of plasmid 104 used for base editing by CRISPR/dCas9-AID. The expression of dCas9-AID is under the control of IPTG inducible promoter pTac. The sgRNA expression cassette consists of a strong constitutive promoter pCI, a 20-bp guide sequence (complementary region for specific DNA binding), a hairpin for dCas9 binding, a transcription terminator derived from *Streptococcus pyogenes*, and a rrnB terminator.


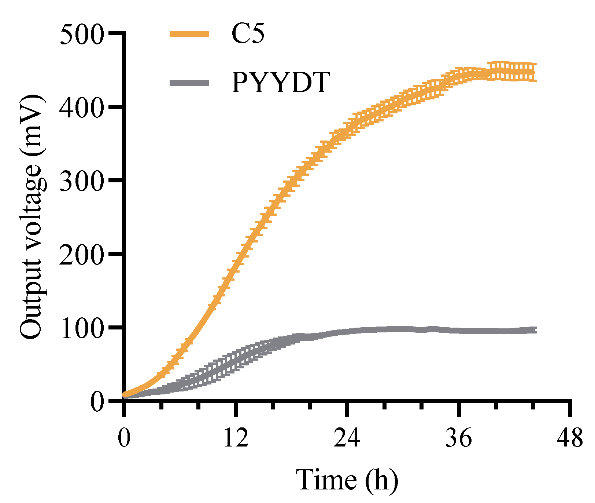


**Supplementary Figure 2.** The output voltage curve of the C5 and PYYDT strains in the MFCs. The error bars (mean ± SD) were derived from duplicate experiments for each strain.


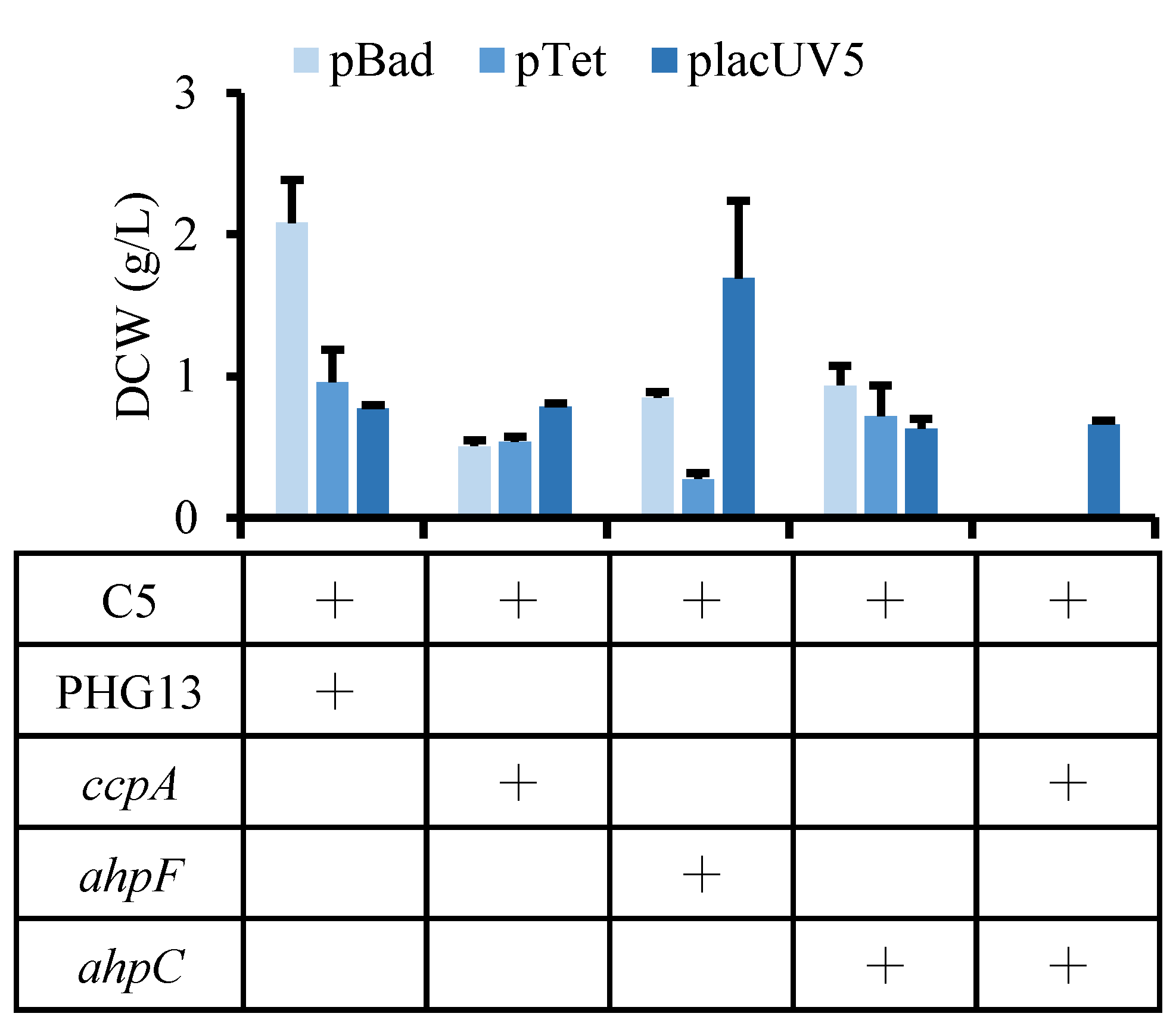


**Supplementary Figure 3.** Dry cell weight (DCW) of the engineered strains overexpressing the selected genes. The error bars (mean ± SD) were derived from triplicate experiments for each strain.

**
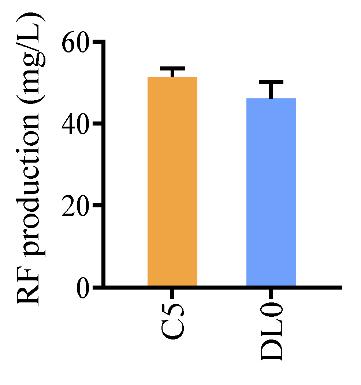
**

**Supplementary Figure 4.** RF production of the C5 and DL0 strains. The error bars (mean ± SD) were derived from triplicate experiments for each strain.

**
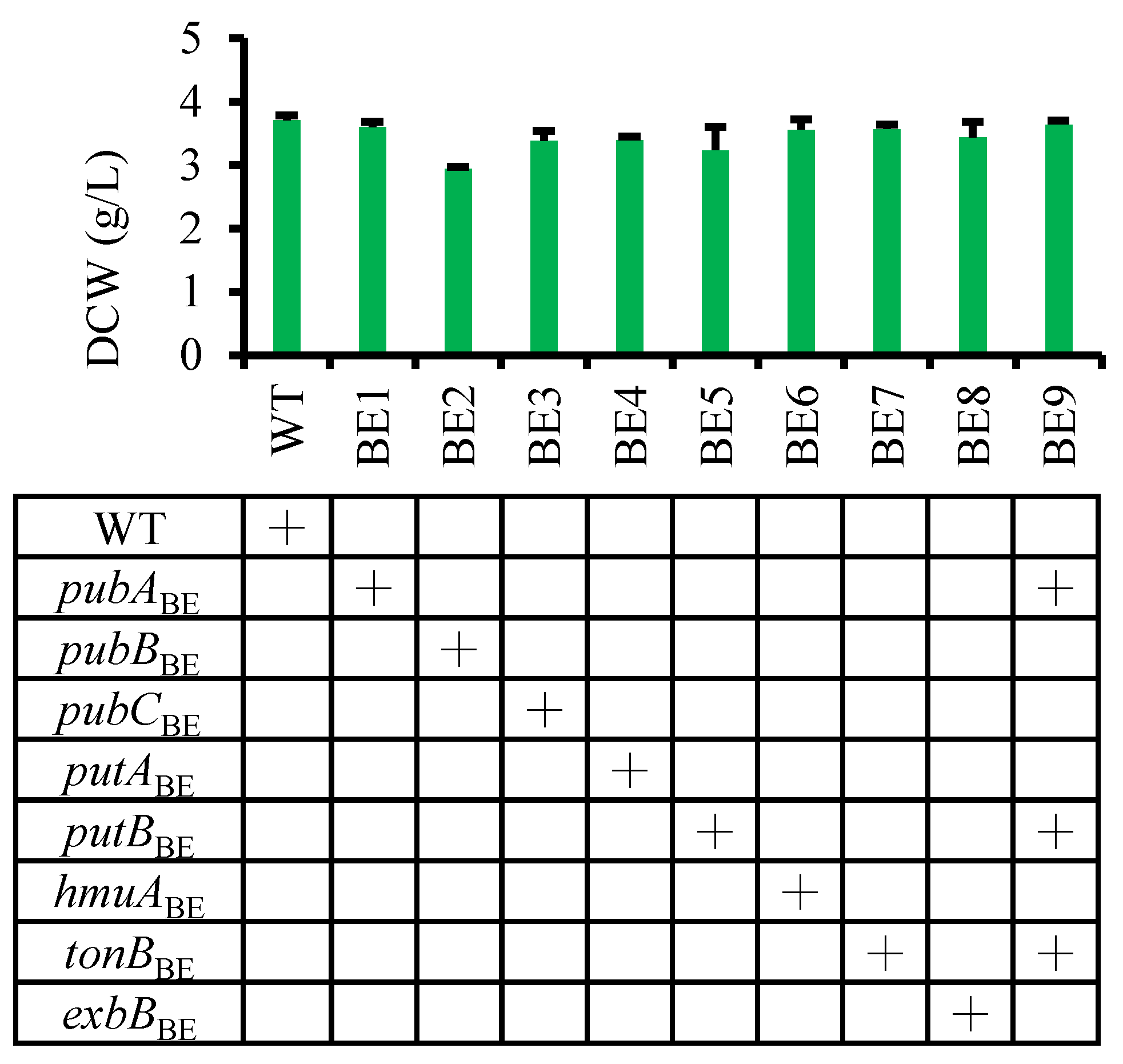
**

**Supplementary Figure 5.** Dry cell weight (DCW) of engineered strains that inactivated the selected genes. The error bars (mean ± SD) were derived from triplicate experiments for each strain.

**Supplementary Table 1.** Primer sequences used for amplifying genes from *S. oneidensis* genome.

| **Primer** | **Sequences (5’-3’)** |
| --- | --- |
| *ccpA*-F | CCGGGCATATGACAAAACTCACCGCT |
| *ccpA*-R | CCGGGGCTAGCTTATTTTGCTCCTGTCGCAA |
| *ahpF*-F | CCGGCCATATGTTAGATGCGAATTTAAAAAATCAAC |
| *ahpF*-R | CCGCCGCTAGCTTAAGCCGCTTTAGTGTCTG |
| *ahpC*-F | CCGGCCATATGACTCAATCAATTATCAACAGC |
| *ahpC*-R | CCGGCGCTAGCTTAGATTTTGCCAACAAGGT |

Note: Restriction sites are underlined.

**Supplementary Table 2.** Primer sequences used for annealing of sgRNAs.

| **Primer** | **Sequences (5’-3’)** |
| --- | --- |
| *tonB*-F | TTGCccaacctacatacccgcgca |
| *tonB*-R | AAACtgcgcgggtatgtaggttgg |
| *pubC*-F | TTGCgcaggctgccaatcgccatt |
| *pubC*-R | AAACaatggcgattggcagcctgc |
| *pubB*-F | TTGCaccaaaactatgccaagttt |
| *pubB*-R | AAACaaacttggcatagttttggt |
| *exbB*-F | TTGCcagcaggatttactggcccg |
| *exbB*-R | AAACcgggccagtaaatcctgctg |
| *pubA*-F | TTGCactcaggtgaattacaacgc |
| *pubA*-R | AAACgcgttgtaattcacctgagt |
| *putA*-F | TTGCtcagctcacttttggctgga |
| *putA*-R | AAACtccagccaaaagtgagctga |
| *putB*-F | TTGCtcaatcagctttgaaccaaa |
| *putB*-R | AAACtttggttcaaagctgattga |
| *hmuA*-F | TTGCgttcagaagagtgctgaaca |
| *hmuA*-R | AAACtgttcagcactcttctgaac |

**Supplementary Table 3.** Specific gene expression in the C5 strain compared with that in the PYYDT strain.

| **Gene** | **ID** | **C5 vs. PYYDT (D4)** | | | | **C5 vs. PYYDT (D12)** | | |
| --- | --- | --- | --- | --- | --- | --- | --- | --- |
|  |  | **Log_2_ (fold change)** | ***q* value** | **significance** | **Log_2_ (fold change)** | | ***q* value** | **significance** |
| *mtrA* | SO_1777 | 1.3292 | 6.23E-127 | True | 0.042087 | | 1.33E-07 | False |
| *mtrB* | SO_1776 | 1.5116 | 0 | True | 0.22115 | | 5.40E-28 | False |
| *mtrC* | SO_1778 | 1.8209 | 0 | True | 0.81872 | | 1.07E-115 | False |
| *ribA* | SO_2831 | -0.18331 | 0.005169 | False | 1.0124 | | 8.22E-13 | True |
| *ribD* | SO_3469 | -0.051973 | 1.45E-05 | False | -0.42683 | | 0.20934 | False |
| *ribE* | SO_3466 | 0.00021218 | 7.05E-07 | False | 0.27672 | | 9.38E-07 | False |
| *ribC* | SO_2296 | 0.15913 | 0.0080819 | False | 0.36948 | | 0.017316 | False |
